# Direct and extended intergenerational contact and young people's attitudes towards older adults

**DOI:** 10.1111/bjso.12146

**Published:** 2016-06-02

**Authors:** Lisbeth Drury, Paul Hutchison, Dominic Abrams

**Affiliations:** ^1^ University of KentUK; ^2^ London Metropolitan UniversityUK

**Keywords:** intergroup contact, extended contact, intergroup anxiety, ageing anxiety, ingroup norms, ageism

## Abstract

Research suggests that positive intergenerational contact can improve young people's attitudes towards older adults. However, today's age‐segregated society may not provide ample opportunities for positive contact between younger and older adults to occur on a regular basis. In three studies, we investigated whether the positive attitudinal outcomes associated with direct contact might also stem from a more indirect form of intergenerational relationship: extended contact. In Study 1 (*N *=* *70), extended contact was associated with more positive attitudes towards older adults even when controlling for direct intergenerational contact (contact frequency and contact quality). In Study 2 (*N *=* *110), the positive effects of direct and extended contact on young people's age‐related attitudes were mediated by reductions in intergroup anxiety and ageing anxiety. The mediational effects of intergroup anxiety were replicated in Study 3 (*N *=* *95) and ingroup norms additionally emerged as a mediator of the positive effects of extended contact on young people's attitudes towards older adults. Discussion focuses on the implications for strategies aimed at tackling ageism.

## Background

Demographic ageing is a recent social phenomenon in most developed countries (Kinsella & He, 2009). Medical advancements and improvements in health care and living standards mean that people are living longer than in previous generations, and this trend is set to accelerate over the next 30–40 years (Kinsella & He, 2009). In the United States (US), the number of people aged 65 or over is expected to increase over the next three decades from 35.9 million (13% of the population) to nearly 70 million (20% of the population). Similar increases in the number of older adults are expected in most developed nations (Kinsella & He, 2009). Reflecting this historically unprecedented demographic shift, a United Nations (2007) report estimated that by 2,047 the number of older people worldwide would outstrip the number of young people for the first time.

While longer life expectancies are a positive outcome, the expanding older population may face challenges such as increased negative attitudes towards older people (Nelson, 2005; North & Fiske, 2012). In a US survey, nearly 80% of respondents aged 60 years or over reported having been discriminated against due to their age (Palmore, 2001), and a European survey found that ageism was the most commonly experienced type of discrimination, ahead of discrimination based on gender, ethnicity, disability, religion or sexual orientation (Abrams, Eilola, & Swift, 2009). Given the prevalence of ageism and the rapidly ageing population, there is an urgent need to understand factors that lead to the development of age‐related attitudes in young people, so that it may be possible to reduce ageism.

The present research used contact theory (Allport, 1954) as a framework to examine young people's attitudes towards older adults. We conducted three studies to investigate the relationships between young people's age‐related attitudes and their prior contact with older adults. The aim is to investigate whether the positive attitudinal outcomes associated with direct intergenerational contact might also stem from a more indirect form of intergenerational relationship: extended contact (Wright, Aron, McLaughlin‐Volpe, & Ropp, 1997).

### Intergenerational contact and attitudes towards older people

The contact hypothesis (Allport, 1954) maintains that contact with outgroup members can, under certain conditions, reduce prejudice. Allport (1954) proposed that intergroup attitudes would be improved when individuals from opposing groups are united within contexts that allow both parties equal status, where they cooperate on tasks with common goals, and have the support of relevant institutions and authorities that create norms of acceptance. However, a meta‐analysis of over 500 laboratory and field studies (Pettigrew & Tropp, 2006) found that while these prerequisite conditions enhanced the prejudice‐reducing effects of contact, they were not essential, and contact alone reduced prejudice.

The majority of contact studies have focused on intercultural contact (e.g., contact based on racial or ethnic group memberships: for a review, see Pettigrew & Tropp, 2006). However, contact is also effective at reducing ageism (e.g., Allan & Johnson, 2009; Caspi, 1984; Meshel & McGlynn, 2004). Caspi (1984) found that children experiencing daily contact with older adults at preschool expressed more positive attitudes towards older people generally and could discern more differences in the ages of older individuals, as compared to children who had no such contact. Likewise, Allan and Johnson (2009) found that young people who regularly interacted with older adults at work expressed more positive attitudes towards older people as a whole, whereas those living with older relatives had more ageist attitudes. Allan and Johnson suggested this could be attributed to differences in the quality of contact experienced at work compared to contact with older relatives at home: contact at work is likely to be with competent older individuals, whereas contact at home is more likely to be with dependent older adults.

The importance of investigating not only the frequency of intergenerational contact but also its quality has been highlighted in several studies (e.g., Bousfield & Hutchison, 2010; Hutchison, Fox, Laas, Matharu, & Urzi, 2010; Schwartz & Simmons, 2001). For example, Schwartz and Simmons (2001) found no relationship between the frequency of intergenerational contact and age‐related attitudes in a sample of college students, whereas those reporting good quality contact had less ageist attitudes. Bousfield and Hutchison (2010) reported similar findings in a survey of university students and additionally found that good quality contact was associated with more positive behavioural intentions towards older adults. This included intentions to spend more time with older adults, help them, and make donations to charities for older people.

While these examples suggest that intergenerational contact can reduce ageism in young people, today's age‐segregated society may not provide ample opportunities for positive and meaningful contact between younger and older individuals to occur on a regular basis (Hagestad & Uhlenberg, 2005). Despite there being more older adults today than at any point in history, changes in moral and political values along with family breakdowns and advances in social media technology mean that people interact primarily with same‐age peers from an early age (Peacock & Talley, 1984). Likewise, social norms discouraging intergenerational relationships may reduce the willingness of both young and older individuals to interact with members of different age groups (Nelson, 2005). Reflecting this, a survey of over 2000 British people confirmed that less than one‐third of over 70‐year‐olds had a friend under the age of 30 and less than one‐third of under 30‐year‐olds had a friend over the age of 70 (Abrams *et al*., 2009). These examples suggest the existence of an increasingly age‐segregated society, which provides the potential for intergenerational misunderstandings leading to the development of ageist attitudes (Abrams *et al*., 2009).

A unique aspect of ageism that adds further complexity to prejudice reduction is that, unlike other outgroups, young people will themselves become members of the older generation. This pending group transition presents unique challenges as young adults may harbour anxieties about their own ageing (Lasher & Faulkender, 1993) and more generally about coming into contact with older adults (Bousfield & Hutchison, 2010; Hutchison *et al*., 2010). This may go some way towards explaining why contact's reduction of ageism is typically smaller in magnitude than its reduction of other types of prejudice (Pettigrew & Tropp, 2006). With these issues in mind, in the present research, we investigated whether the positive attitudinal outcomes associated with direct intergenerational contact might also arise from extended contact (Wright *et al*., 1997).

### Extended contact

A relatively recent development in contact theory is the extended contact hypothesis (Wright *et al*., 1997), which holds that knowing that other ingroup members have positive relationships with outgroup members can promote more positive outgroup attitudes. In the present context, this would mean that knowing that their same‐age peers have positive relationships with older individuals might be sufficient to improve young people's attitudes towards older adults as a whole.

Extended contact has been found to predict more positive attitudes towards a host of stigmatized and marginalized groups (e.g., Eller, Abrams, & Gómez, 2012; Gómez, Tropp, & Fernández, 2011; Hutchison & Rosenthal, 2011; Wright *et al*., 1997), but the ability of extended contact to reduce ageism has yet to be empirically established (see Christian, Turner, Holt, Larkin, & Cotler, 2014; Paolini, Hewstone, & Cairns, 2007). The lack of research in this area is surprising in the light of research suggesting that the generation gap in many developed countries is as wide as it has been since the 1960s (Pew Research Center, 2009) and opportunities for the formation of positive relationships between young and older adults are becoming increasingly limited (Abrams *et al*., 2009). Extended contact may be especially useful in the current social climate as it implies that direct intergenerational relationships may not be essential for the positive outcomes associated with contact to be realized.

Another advantage of extended contact is that it allows individuals to experience intergroup relationships while avoiding the anxieties often associated with direct intergroup encounters (Stephan & Stephan, 1985). It can also be useful in preparing members of opposing groups for future direct contact (Eller *et al*., 2012). Thus, it seems both worthwhile and timely to explore whether extended contact might have similar positive outcomes in the context of young people's attitudes towards older adults.

### Mediators of direct and extended contact

Having confirmed that direct and extended contact can reduce prejudice, recent research has investigated mediating variables (for a review, see Pettigrew & Tropp, 2008). One such variable is intergroup anxiety (Stephan & Stephan, 1985), which refers to the negative affect often experienced during or in anticipation of intergroup encounters. Individuals may worry that interactions with outgroup members will lead to rejection, embarrassment or misunderstanding, influencing the development of prejudice (Plant & Devine, 2003; Stephan & Stephan, 1985). Indeed, intergroup anxiety predicts a range of undesirable outcomes including negative outgroup attitudes and avoidance of outgroup members (e.g., Esses & Dovidio, 2002; Stephan, Diaz‐Loving, & Duran, 2000).

Several studies have shown that good quality intergroup contact can reduce intergroup anxiety and indirectly reduce different types of prejudice (for a review, see Pettigrew & Tropp, 2008) including ageism (Bousfield & Hutchison, 2010; Hutchison *et al*., 2010). For example, Bousfield and Hutchison (2010) found that good quality contact with older adults reduced young people's concerns about impending intergenerational encounters, which in turn improved their attitudes towards older adults as a whole (see also Hutchison *et al*., 2010). However, the links between extended contact, intergroup anxiety, and the age‐related attitudes of young people have not yet been explored.

Another form of anxiety associated with ageism is ageing anxiety, which Lasher and Faulkender (1993, p. 247) defined as ‘the combined concern and anticipation of losses centred around one's own ageing process’. Thus, whereas intergroup anxiety arises due to concerns about the anticipated outcomes of intergroup interactions, ageing anxiety is thought to arise from concerns related to the negative aspects of one's personal ageing. This includes concerns about the anticipated loss of one's independence, close friends and relatives, physical and mental health and, ultimately, one's very existence (Lasher & Faulkender, 1993). Consequently, older adults may present a threat to young people by reminding them of the inevitable consequences of their own ageing (Greenberg, Schimel, & Martens, 2002). Greenberg *et al*. (2002) proposed that individuals cope with the anxiety induced by such threats by denigrating entities that serve as reminders of their own fate (see also Martens, Greenberg, Schimel, & Landau, 2004; Nelson, 2005). Consistent with this reasoning, several studies have found a positive correlation between ageing anxiety and ageism (Allan & Johnson, 2009; Allan, Johnson, & Emerson, 2014; Boswell, 2012; Harris & Dollinger, 2001).

Research has shown that intergenerational contact can reduce ageing anxiety and indirectly reduce ageism. Allan and colleagues (Allan & Johnson, 2009; Allan *et al*., 2014) found that frequent intergenerational contact was associated with less ageism in young people and this reduction in ageism was mediated by a decrease in ageing anxiety: the more contact young people have with older adults, the less anxious they are about their own ageing, and the less ageist they are. However, attempts to replicate these effects have not always been successful (Bousfield & Hutchison, 2010; Hutchison *et al*., 2010). For example, Bousfield and Hutchison (2010) found that reduced intergroup anxiety mediated the positive effect of good quality intergenerational contact on young people's age‐related attitudes but ageing anxiety did not. Thus, further tests of the potential of intergenerational contact to reduce ageing anxiety and ageism are required.

As well as providing such a test the present research additionally examined the relationship between extended contact and ageing anxiety for the first time. It is conceivable that, like direct intergenerational contact, extended contact might help to reduce some of the concerns that young people often have about their own ageing (e.g., Eshbaugh, Gross, & Satrom, 2010) and therefore indirectly reduce ageism.

A further variable that has been shown to mediate the positive effects of contact is ingroup norms (e.g., Davies, Wright, Aron, & Comeau, 2013). Ingroup norms represent the shared understandings among ingroup members about appropriate group‐based actions, thoughts, values, and beliefs (Cialdini, Kallgren, & Reno, 1991). Ingroup norms are particularly important in extended contact situations and are an integral part of the process (Wright *et al*., 1997). Knowing that ingroup members have positive relationships with outgroup members can increase the acceptability of such relationships by making them seem more widespread and familiar, thus indirectly reducing prejudice (e.g., Cameron, Rutland, Hossain, & Petley, 2011; De Tezanos‐Pinto, Bratt, & Brown, 2010; Turner, Hewstone, Voci, & Vonofakou, 2008). Along these lines, Turner *et al*. (2008) found that extended contact between White British students and South Asians in the United Kingdom was associated with more positive outgroup attitudes, and this relationship was mediated by increased norms of positive intercultural relationships. Similarly, Cameron *et al*. (2011) found that ingroup norms mediated the positive influence of extended contact on British adolescents’ attitudes towards Asians. These examples suggest that ingroup norms are important for young people (see also Schofield & Eurich‐Fulcer, 2001) and therefore may influence their inhibitions about, and ultimately their attitudes towards, older adults. However, no research to date has assessed the role of ingroup norms in intergenerational contact situations.

A final mediating variable investigated in the present research is self‐disclosure, which refers to the voluntary sharing of intimate or personal information (Miller, 2002). Self‐disclosure is integral to the formation and maintenance of positive interpersonal relationships (Altman & Taylor, 1973; Reis & Shaver, 1988). Receiving intimate disclosure increases interpersonal trust and liking of the discloser (Collins & Miller, 1994) and is likely to be reciprocated, leading to mutual interpersonal attraction (Laurenceau, Barrett, & Rovine, 2005).

Self‐disclosure is also important for the development of positive intergroup relationships and the reduction of prejudice (e.g., Dovidio *et al*., 1997; Ensari & Miller, 2002; Turner, Hewstone, & Voci, 2007). For example, Ensari and Miller (2002) found that self‐disclosure by a typical outgroup member during a cooperative intergroup activity not only improved liking of the discloser, but also improved attitudes towards the outgroup as a whole. Similarly, Turner *et al*. (2007) found that the more interethnic friendships that White British schoolchildren had, the less prejudiced they were, and this association was mediated by an increased willingness to self‐disclosure to an outgroup member. In the same study, the positive relationship between extended contact and outgroup attitudes was also explained by increased willingness to self‐disclose to an outgroup member. These findings confirm that self‐disclosure is important for the development of positive intergroup relationships in young people and prejudice reduction (Dovidio *et al*., 1997). However, the role of self‐disclosure in direct or extended intergenerational contact situations has yet to be empirically established (but see Harwood, Hewstone, Paolini, & Voci, 2005).

### Overview of the present research

The present research used contact theory (Allport, 1954) as a framework to investigate young people's attitudes towards older adults. Study 1 examined the relationships between the age‐related attitudes of young people and three different types of intergenerational contact: contact frequency, contact quality, and extended contact. Studies 2 and 3 developed the analysis to additionally investigate the psychological mechanisms through which direct and extended contact reduce ageism.

## STUDY 1

### Method

#### Participants

Seventy students at a London university participated in the study. Thirty‐eight were female and 32 were male. Ages ranged from 17 to 25, with a mean age of 21.16 years (*SD* = 2.12).

#### Materials and procedure

Students were approached on a university campus and invited to take part in a study on ‘elderly people in modern society’. Those who agreed were handed a questionnaire containing all instructions and measures, which were presented in the same order as described below. It was explained to participants that the term ‘elderly’ referred to people aged 65 years or over, while ‘contact’ was defined as ‘interactions with elderly individuals – for example at work, socially, in the neighbourhood’.

##### Contact measures

Contact frequency was assessed by asking participants to indicate how often they had contact with elderly individuals on a scale ranging from 1 (*very rarely*) to 5 (*very often*). Contact quality was assessed by asking participants to rate the quality of their previous intergenerational contact using three 5‐point scales with endpoints labelled: unpleasant–pleasant, voluntary–involuntary, and bad quality–good quality. The items were combined (averaged) to form a single contact quality score (Cronbach's α* *= .73). Extended contact was assessed by asking participants to indicate how many of their close friends have positive relationships with older adults on a scale ranging from 1 (*none at all*) to 5 (*very many*). Items were scored such that higher scores indicate more contact frequency, better quality contact, and more extended intergenerational relationships, respectively.

##### Attitude measure

Participants indicated their attitudes towards older adults using six 5‐point scales with endpoints labelled: warm–cold, negative–positive, friendly–hostile, suspicious–trusting, respect–contempt, and admiration–disgust (adapted from Wright *et al*., 1997). Participants were informed that their responses should reflect their feelings about older adults in general, not those with whom they or their close friends have contact. Responses were scored such that higher scores indicate a more positive attitude (α* *= .89).

## Results

Table 1 displays the means and standard deviations for the measures as well as their intercorrelations. As shown in that table, both contact quality and extended contact were positively correlated with young people's attitudes towards older adults, whereas contact frequency and attitudes were not correlated. Although not indicated in Table 1, gender or age of the participants were not correlated with any other variables, all *r*s < −.19, all *p*s > .12.

**Table 1:** Means, standard deviations, and correlations between the variables for Studies 1, 2, and 3

Measures	*M*	*SD*	2	3	4	5	6	7	8
Study 1									
1. Contact frequency	2.41	1.23	−.09	.03	.08				
2. Contact quality	2.92	1.12		−.08	.38**				
3. Extended contact	3.36	1.14			.34**				
4. Attitudes	3.46	0.78							
Study 2									
1. Contact frequency	4.52	1.86	.28**	.32**	.15	−.14	−.21*		
2. Contact quality	5.20	1.11		.15	.39***	−.29***	−.33***		
3. Extended contact	3.67	1.60			.35***	−.34***	−.37***		
4. Attitudes	5.64	0.89				−.42***	−.41***		
5. Intergroup anxiety	2.86	1.33					.28**		
6. Ageing anxiety	3.47	1.17							
Study 3									
1. Contact frequency	4.20	2.43	.18	.48***	.16	−.34**	−.19	.18	.27**
2. Contact quality	4.75	1.01		.19	.43***	−.51**	−.23*	.33**	.41***
3. Extended contact	1.96	0.79			.22*	−.24*	−.21*	.39***	.35**
4. Attitudes	5.10	1.07				−.51**	−.22*	.35**	.36**
5. Intergroup anxiety	3.17	1.12					.32**	−.24*	−.40***
6. Ageing anxiety	4.91	1.34						−.25*	−.16
7. Ingroup norms	3.97	1.11							.48***
8. Self‐disclosure	4.27	1.37							–

Note

*p <* *.05; **p <* *.01; ***p <* *.001. Scores on all measures range from 1–5 in Study 1 and 1–7 in Study 2. In Study 3, scores on all measures range from 1–7 except the extended contact scores, which range from 1–5.

Next, we conducted a multiple linear regression analysis to assess the extent to which the three contact variables predict attitudes (see Table 2). Gender and age were included as control variables. The regression equation was significant, *F* (5, 64) = 5.18, *p *<* *.001, *R* ^2^ = .29. Replicating the correlation results, both contact quality, *B *=* *.39, *SE* = .11, *t *=* *3.67, *p *<* *.001, and extended contact, *B *=* *.38, *SE* = .11*, t *=* *3.59, *p *=* *.001, were positively associated with attitudes, whereas contact frequency was not significantly associated with attitudes, *B *=* *.14, *SE* = .11*, t *=* *1.25, *p *=* *.22.

**Table 2:** Summary of multiple regression analyses examining the effects of contact variables on attitudes towards older adults in Studies 1, 2, and 3

	Study 1			Study 2			Study 3		
	B	*SE*	*t*	B	*SE*	*t*	B	*SE*	*t*
Independent variable									
Contact frequency	.14	.11	1.25	−.04	.10	−0.44	.02	.11	0.20
Contact quality	.39	.11	3.67***	.35	.09	3.83***	.39	.10	3.78***
Extended contact	.39	.11	3.59***	.31	.09	3.27**	.14	.12	1.26
Control variables									
Gender	.11	.22	0.53	.04	.19	0.21	−.16	.20	−0.81
Age	.12	.11	1.12	−.02	.09	−0.16	.10	.11	0.92
Employment status							.06	.28	0.20
R	.54			.49			.47		
*R* ^2^	.29			.24			.22		

Note

**p <* *.01; ***p <* *.001. Gender 1 = *Male*, 2 = *Female*. Employment status 1 = *Employed,* 2 = *Student*.

## Discussion

Study 1 investigated the relationships between different types of intergenerational contact and young people's attitudes towards older adults. The results suggest that having frequent contact with older adults may not be sufficient to reduce ageism. Instead, the data suggest that it is the perceived quality of intergenerational contact that has the potential to reduce ageism. Finding that contact quality is associated with less ageism but contact frequency is not is consistent with previous studies (Bousfield & Hutchison, 2010; Hutchison *et al*., 2010; Schwartz & Simmons, 2001). Extending previous research, the present results additionally suggest that direct contact may not even be necessary to reduce ageism: simply knowing that their same‐age peers have positive intergenerational relationships may be sufficient to improve young people's attitudes towards older adults.

We conducted a second study to test the robustness of these effects and to additionally examine intergroup anxiety and ageing anxiety as potential mediators of the positive associations between both direct and extended intergenerational contact and young people's attitudes towards older adults.

## STUDY 2

### Method

#### Participants

Participants were 110 psychology students at a London university. Sixty‐eight were female, 41 were male, and one participant did not indicate their gender. Ages ranged from 18 to 25 with a mean age of 21.21 years (*SD* = 2.12). None had participated in Study 1.

#### Materials and procedure

The study was conducted in a lecture hall as part of a scheduled teaching session. All instructions and measures were presented in a questionnaire in the same order as described below. As in Study 1, it was explained to participants that the term ‘elderly’ referred to people aged 65 years or over and ‘contact’ referred to ‘time spent interacting with elderly people’.

##### Contact measures

Contact frequency was assessed by asking participants to indicate how often they had contact with elderly individuals on a scale ranging from 1 (*very rarely*) to 7 (*very often*). Contact quality was assessed using three 7‐point scales with the same endpoint labels as used in Study 1 (α* *= .71). Extended contact was assessed by asking participants to indicate how many of their close friends had positive relationships with older adults on a scale ranging from 1 (*none at all*) to 7 (*very many*). Responses were scored such that higher values indicate more contact frequency, better quality contact, and more extended contact, respectively.

##### Intergroup anxiety measure

Intergroup anxiety was assessed by asking participants how they would feel interacting with a typical elderly person using three pairs of bipolar adjectives separated by a 7‐point scale. The adjective pairs were as follows: tense–relaxed, calm–nervous, and stressed–unstressed. Participants were informed that their responses should reflect their feelings about interacting with a typical older adult and not necessarily those older individuals with whom they have contact. A similar measure has been used in several previous studies (e.g., Bousfield & Hutchison, 2010). Responses were scored such that higher scores indicate more intergroup anxiety (α* *= .78).

##### Ageing anxiety measure

Ageing anxiety was assessed using four items asking participants how they feel about personally ageing: ‘I am worried that I will lose my independence when I am old’, ‘I am relaxed about getting old’, ‘I am concerned that my mental abilities will suffer when I am old’, and ‘I do not want to get old because it means I am closer to dying’. These items were adapted from measures used in previous research (e.g., Lasher & Faulkender, 1993). Responses were recorded on a 7‐point scale (1 = *strongly disagree*, 7 = *strongly agree*). Higher scores indicate more ageing anxiety (α* *= .91).

##### Attitude measure

Participants indicated their attitudes towards older people on the same six pairs of bipolar adjectives as used in Study 1 using a 7‐point scale. The items were scored such that a higher score indicates a more positive attitude (α* *= .81).

## Results

Table 1 displays the means and standard deviations for the measures along with their intercorrelations. As in Study 1, both contact quality and extended contact were positively correlated with young people's attitudes towards older adults, whereas contact frequency and attitudes were not significantly correlated. Likewise, both contact quality and extended contact were negatively correlated with intergroup anxiety, but contact frequency was not. All three contact variables were negatively correlated with ageing anxiety, and both intergroup anxiety and ageing anxiety were negatively correlated with attitudes. Although not indicated in Table 1, gender or age was not correlated with any of the other variables, all *r*s < .18, *p*s > .11.

As in Study 1, we used multiple regression analysis (see Table 2) to assess the extent to which the three contact variables predict attitudes (controlling for gender and age). The regression equation was significant *F* (5,102) = 6.26, *p *<* *.001. Replicating the results from Study 1, contact quality, *B *=* *.35, *SE* = .01, *t *=* *3.83, *p *<* *.001, and extended contact, *B *=* *.31, *SE* = .01, *t *=* *3.27, *p *=* *.001, were positively associated with attitudes, whereas contact frequency was not associated with attitudes, *B *=* *−.04, *SE* = .10, *t *=* *−0.44, *p *=* *.66.

### Mediation analyses

We followed the procedures outlined by Preacher and Hayes (2008) to assess whether intergroup anxiety and/or ageing anxiety mediated the positive association between intergenerational contact (contact quality and extended contact) and young people's age‐related attitudes. To this end, we used the Indirect Macro for SPSS (Preacher & Hayes, 2008), which uses bootstrapping techniques to estimate the total and direct effects of a predictor variable on an outcome variable as well as the indirect effects through one or more mediator variables. These analyses have the advantage of greater statistical power without assuming multivariate normality in the sampling distribution and are more appropriate than alternative techniques (e.g., structural equation modelling) when the sample size is relatively small (Hayes, 2013). In these analyses, an indirect effect (*PE*) is significant if the bias‐corrected 95% confidence interval (BC CI) does not include zero.

#### Mediation of the contact quality – attitudes relationship

In this analysis, intergroup anxiety and ageing anxiety were investigated as mediators of the association between contact quality and attitudes. As well as gender and age, contact frequency and extended contact were included in the model as covariates. The total effect of contact quality on attitudes was significant, *B *=* *.35, *SE* = .09, *t *=* *3.82, *p *<* *.001, as was the direct effect, *B *=* *.23, *SE* = .09, *t *=* *2.46, *p *=* *.02. The total indirect effect through intergroup anxiety and ageing anxiety was significant, *PE* = .12, *SE* = .06, BC CI [.026, .263], as were the specific indirect effects through intergroup anxiety, *PE* = .06, *SE* = .04, BC CI [.007, .179], and ageing anxiety, *PE *= .06, *SE* = .04, BC CI [.004, .175]. This confirms that intergroup anxiety and ageing anxiety both mediate between contact quality and young people's attitudes towards older adults.

#### Mediation of the extended contact – attitudes relationship

In this analysis, intergroup anxiety and ageing anxiety were investigated as mediators of the association between extended contact and attitudes. Contact frequency, contact quality, age, and gender were included as covariates. The total effect of extended contact was significant, *B *=* *.31, *SE* = .09, *t *=* *3.27, *p *=* *.002, but the direct effect was not, *B *=* *.16, *SE* = .09, *t *=* *1.64, *p *=* *.10. The total indirect effect through the two anxiety variables was significant, *PE *= .15, *SE* = .07, BC CI [.039, .326], as were the specific indirect effects through intergroup anxiety, *PE *= .07, *SE* = .04, BC CI [.014, .192], and ageing anxiety, *PE* = .07, *SE* = .05, BC CI [.002, .204]. This confirms that intergroup anxiety and ageing anxiety also mediate between extended contact and young people's age‐related attitudes.

## Discussion

Study 2 investigated the relationships between different types of intergenerational contact and young people's age‐related attitudes and additionally examined intergroup anxiety and ageing anxiety as potential mediators of contact's reduction of ageism. Finding that contact quality and extended contact were associated with more positive attitudes towards older people, whereas contact frequency was not, is consistent with the results from Study 1 and several prior studies (Bousfield & Hutchison; Hutchison *et al*., 2010; Schwartz & Simmons, 2001). Extending previous research, the results from Study 2 additionally show that the positive effects of both contact quality and extended contact on young people's age‐related attitudes are mediated by reduced intergroup anxiety and ageing anxiety. This suggests that the effects of experiencing indirect intergenerational relationships may be similar to those associated with experiencing good quality direct contact with older individuals. More specifically, the results suggests that when young people experience good quality personal contact with older adults or when they experience positive indirect intergenerational relationships within their close social environment, they feel less anxious about possible future intergenerational encounters and their own ageing and thus are less ageist.

A limitation with Studies 1 and 2 concerns the samples used. Both studies were conducted with student samples, and it is possible that students may have different experiences of contact with older adults as compared to non‐students, especially since students are more likely to come into contact with competent older individuals on a regular basis (e.g., professors, mature students). Thus, it is possible that students and non‐students may differ in terms of the quality of contact they experience with older adults as well as the number and nature of indirect relationships they may be aware of. For this reason, we conducted a third study with a more diverse sample.

In addition, in Study 3, we replaced the single‐item extended contact measure used in Studies 1 and 2 with a multi‐item measure that has been used in previous contact studies (Turner *et al*., 2008). Finally, as well as intergroup anxiety and ageing anxiety, in Study 3, we additionally examined ingroup norms and self‐disclosure as potential mediating variables. These variables have been shown in previous research to mediate the positive effects of direct and extended contact on outgroup attitudes (Davies *et al*., 2013; Turner *et al*., 2007, 2008), but not in the context of intergenerational contact.

## STUDY 3

### Method

#### Participants

The sample consisted of 95 participants, 61 males and 34 females. Ages ranged from 18 to 30 years (*M* = 24.52, *SD* = 3.29). Participants were asked to indicate their current primary occupation: 82% were in either full‐time or part‐time employment, and 18% were students.

#### Materials and procedure

Participants were recruited via Amazon's Mechanical Turk online tool and received $0.40 for completing a survey on ‘friendships’. The terms ‘elderly’ and ‘contact’ were defined as in Studies 1 and 2.

##### Contact measures

As in Study 2, contact frequency was assessed by asking participants to indicate how often they had contact with elderly individuals on a scale ranging from 1 (*very rarely*) to 7 (*very often*). Contact quality was assessed using three 7‐point scales with the same endpoint labels as used in Studies 1 and 2 (α* *= .65). Extended contact was assessed using four items adapted from measures used in prior research (Turner *et al*., 2008): ‘How many of your friends in your age group have friends who are elderly?’, ‘How many of your very best friends in your age group have friends that are elderly?’, ‘How many of your family members in your age group have friends who are elderly?’ (1 = *none,* 5 = *over ten*), and ‘How many people in your age group do you know who have friends who are elderly’ (1 = *none,* 5 = *most*). The items were combined to form a single extended contact score (α* *= .82). Higher scores indicate more extended contact.

##### Anxiety measures

Intergroup anxiety (α* *= .86) and ageing anxiety (α* *= .80) were assessed using the same measures as in Study 2. Higher scores indicate more intergroup anxiety and ageing anxiety, respectively.

##### Ingroup norms

Ingroup norms was assessed using four items adapted from previous research (Turner *et al*., 2008): ‘Most of your friends (in your age group) would consider it something positive to have elderly people as friends’, ‘Most of your friends (in your age group) would choose to have a friend who is elderly’, ‘People in your age group like elderly people’ (1 = *totally disagree*, 7 = *totally agree*), and ‘Do you think your friends in your age group would be happy to socialize with someone who is elderly?’ (1 = *not at all happy,* 7 = *very happy*). The items were combined to form a single ingroup norms score (α* *= .85). A higher score indicates more positive ingroup norms about friendships with older adults.

##### Self‐disclosure

Self‐disclosure was assessed using four items that were also adapted from previous research (Turner *et al*., 2007). Participants indicated how willing they would be to disclose the following information to an elderly person on a scale ranging from 1 (*definitely not*) to 7 (*definitely*): ‘a self‐relevant problem’, ‘an exciting secret’, ‘their feelings’, and ‘personal information’. The items were averaged to create a single self‐disclosure score (α* *= .89), with higher scores indicating more willingness to self‐disclose.

##### Attitude measure

Participants indicated their attitudes towards older people on the same six pairs of bipolar adjectives as used in Studies 1 and 2 using a 7‐point scale. A higher score indicates a more positive attitude (α* *= .88).

## Results

Table 1 displays the means and standard deviations for the measures along with their intercorrelations. As in Studies 1 and 2, contact quality and extended contact were positively correlated with young people's attitudes towards older people whereas contact frequency was not. In addition, all three contact variables were negatively correlated with intergroup anxiety but only contact quality and extended contact were negatively associated with ageing anxiety. Similarly, contact quality and extended contact were positively associated with ingroup norms about intergenerational relationships but contact frequency was not, and all three contact variables were positively correlated with willingness to self‐disclose to an older adult. As expected, intergroup anxiety and ageing anxiety were associated with more negative attitudes towards older adults, whereas ingroup norms and self‐disclosure were associated with more positive age‐related attitudes. Finally, although not included in Table 1, the demographic variables were not associated with any of the other variables except age, which was positively correlated with contact quality, *r *=* *.23, *p *=* *.026, and self‐disclosure, *r *=* *.21, *p *=* *.039, all other *r*s < .18, all *p*s > .09.

As in Studies 1 and 2 we used multiple regression analysis (see Table 2) to assess the extent to which the different types of contact predict young people's age‐related attitudes (controlling for the demographic variables). The regression equation was significant, *F* (6, 88) = 4.08, *p *=* *.001, *R* ^2^ = .22. Contact quality was positively associated with attitudes towards older adults, *B *=* *.39, *SE* = .11, *t *=* *3.78, *p *<* *.001, whereas extended contact, *B *=* *.14, *SE* = .12, *t *=* *1.26, *p *<* *.211, and contact frequency were not associated with attitudes, *B *=* *.02, *SE* = .11, *t *=* *0.20, *p *=* *.843.

### Mediation analysis

Following the procedures outlined in Study 2, we examined whether intergroup anxiety, ageing anxiety, ingroup norms, and self‐disclosure mediate between both contact quality and extended contact and young people's attitudes towards older adults.

#### Mediation of the contact quality – attitudes relationship

In this analysis, intergroup anxiety, ageing anxiety, ingroup norms, and self‐disclosure were investigated as mediators of the positive association between contact quality and young people's attitudes towards older adults. Contact frequency, extended contact, and the demographic variables were included as covariates. The total effect of contact quality on attitudes was significant, *B *=* *.39, *SE *= .10, *t *=* *3.78, *p *<* *.001, whereas the direct effect was not, *B *=* *.15, *SE *= .11, *t *=* *1.34, *p *=* *.185. The total indirect effect through all four mediator variables was also significant, *PE* = .24, *SE* = .08, *BI CI* [.100, .412], as was the specific indirect effect through intergroup anxiety, *PE* = .17, *SE* = .08, *BI CI* [.052, .353]. However, the specific indirect effects through ageing anxiety, *PE* = .002, *SE* = .02, *BI CI* [−.043, .056], ingroup norms, *PE* = .05, *SE* = .04, *BI CI* [−.004, .149], and self‐disclosure, *PE* = .02, *SE* = .05, *BI CI* [−.050, .136] were not significant. This confirms that intergroup anxiety mediates between contact quality and young people's attitudes towards older adults.

#### Mediation of the extended contact – attitudes relationship

In this analysis intergroup anxiety, ageing anxiety, ingroup norms, and self‐disclosure were investigated as mediators of the positive association between extended contact and young people's age‐related attitudes. Contact frequency, contact quality, and the demographic variables were included as covariates. The total effect of extended contact on attitudes was significant, *B *=* *.24, *SE *= .10, *t *=* *2.32, *p *=* *.023, whereas the direct effect was not, *B *=* *.02, *SE *= .11, *t *=* *0.19, *p *=* *.847. The total indirect effect was also significant, *PE* = .22, *SE *= .07, *BI CI* [.101, .381] as were the specific indirect effects through intergroup anxiety, *PE* = .11, *SE *= .05, *BI CI* [.031, .263], and ingroup norms, *PE* = .09, *SE *= .05, *BI CI* [.006, .213]. However, the specific indirect effect through ageing anxiety, *PE* = −.0002, *SE *= .02, *BI CI* [−.042, .049], and self‐disclosure, *PE* = .01, *SE *= .05, *BI CI* [−.065, .145], were not significant. This confirms that intergroup anxiety and ingroup norms mediate between extended contact and young people's age‐related attitudes.

## Discussion

Study 3 investigated the relationships between different types of intergenerational contact and young people's age‐related attitudes and additionally examined the potential mediating roles of intergroup anxiety, ageing anxiety, ingroup norms, and self‐disclosure. Like those from Studies 1 and 2, the present results suggest that frequent intergenerational contact alone may not be sufficient to reduce ageism; it is the perceived quality of intergenerational contact and the knowledge that other young people have positive relationships with older adults that has the potential to improve young people's attitudes towards older adults. Moreover, as in Study 2, intergroup anxiety mediated the positive effects of both contact quality and extended contact on young people's age‐related attitudes. However, unlike Study 2, ageing anxiety did not emerge as a significant mediator in Study 3 and neither did self‐disclosure but ingroup norms mediated the effect of extended contact on attitudes. Together, these results suggest that experiencing good quality direct contact with older adults reduces the concerns many young people have about intergenerational encounters, which in turn improves their attitudes towards older adults as a whole. Likewise, knowing that other young people in their close social network have positive relationships with older adults can similarly reduce intergroup anxiety and make such relationships seem more widespread and acceptable, and thus indirectly reduce ageism.

## GENERAL DISCUSSION

The developed world is experiencing a rapid shift towards greater numbers of older people relative to younger people, as average life expectancies continue to increase and birth rates decrease (United Nations, 2007). This significant demographic shift is occurring against a background of research suggesting that ageism is the most common type of prejudice (Abrams *et al*., 2009). This suggests an urgent need to better understand factors that lead to the development of age‐related attitudes in young people, so that it may be possible to reduce ageism.

In three studies, we examined the relationships between different types of intergenerational contact and young people's attitudes towards older adults. Although no significant relationships emerged between the frequency of intergenerational contact and young people's age‐related attitudes, contact quality and extended contact were associated with more positive attitudes towards older people in all three studies. Finding that good quality contact is associated with less ageism but contact frequency is not is consistent with several previous intergenerational contact studies (Bousfield & Hutchison, 2010; Hutchison *et al*., 2010; Schwartz & Simmons, 2001). Likewise, although there is some evidence in the contact literature of a link between contact frequency and prejudice (see Pettigrew & Tropp, 2006), good quality contact typically emerges as the stronger and more reliable predictor of reduced prejudice (e.g., Islam & Hewstone, 1993; Prestwich, Kenworthy, Wilson, & Kwan‐tat, 2008; Tawagi & Mak, 2015). Thus, the present results concur with those in the wider contact literature and suggest that frequent contact with older individuals may not be sufficient to reduce ageism; it is the perceived quality of intergenerational contact that has the greater potential to improve young people's attitudes towards older adults and therefore reduce ageism. The present results are therefore consistent with Allport's (1954) emphasis on the nature of intergroup contact rather than on contact *per se* (see also Amir, 1969).

Although extended contact was associated with less ageism in all three studies, when we tested the unique effects of each type of contact (direct frequency, direct quality, and extended contact) using regression analyses we found that in Studies 1 and 2 both contact quality and extended contact accounted for variance in attitudes over and above the variance accounted for by direct forms of contact, whereas in Study 3, only contact quality emerged as a unique predictor of attitudes. Nevertheless, when meta‐analytically summarized over the three studies, extended contact was associated with less ageism even when controlling for the effects of direct contact.^1^ Therefore, the current research shows that in addition to direct contact between young and older adults, extended intergenerational contact can influence young people's attitudes towards older adults and therefore reduce ageism. This suggests that direct contact with older adults may not even be necessary for the positive attitudinal outcomes of contact to be realized – simply knowing that other young people have positive relationships with older adults may be sufficient to reduce ageism.

One reason why extended contact independently predicted ageism in Studies 1 and Study 2 but not in Study 3 may be due to the samples used. Studies 1 and 2 used student samples, whereas Study 3 used a less homogeneous sample consisting primarily of non‐students. Contact occurring within a university context may provide the contact participants with an additional shared identity (e.g., as students) making the contact appear more normative, whereas contact in other contexts may appear less normative and hence explain the weaker effects of extended contact in Study 3's more diverse sample. This is consistent with Fox and Giles's (1993) proposed model of intergenerational contact, which suggests that contact contexts and/or locations can alter the perceived status of the groups in the contact situation and affect attitudes resulting from contact.

An interesting finding in all three studies is that contact quality and extended contact were associated with the other variables in similar ways while being uncorrelated with each other. In contrast, in intercultural contact studies, contact quality and extended contact are typically positively correlated (e.g., Gómez *et al*., 2011; Hutchison & Rosenthal, 2011) as are direct and indirect friendships (De Tezanos‐Pinto *et al*., 2010; Turner, Tam, Hewstone, Kenworthy, & Cairns, 2013; Turner *et al*., 2007). This suggests that the effects of direct and indirect intergenerational contact may be unique, as they appear to occur independently but have similar attitudinal outcomes. A possible explanation for this finding is that, due to social norms discouraging intergenerational relationships (Nelson, 2005), young adults with intergenerational friends are less likely to tell other young adults about such friendships and similarly they may be less likely to receive information from their peers about their friendships with older adults. In contrast, a young adult with an interethnic friend may be more likely to disclose and even promote this relationship to his or her peers as intercultural friendships are supported by wider societal norms as a progressive way to reduce prejudice towards ethnic minorities (Aboud, Mendelson, & Purdy, 2003). This may explain why contact quality and extended contact are correlated in the wider contact literature but not in intergenerational contact situations.

In Studies 2 and 3, we additionally examined mediating variables. In both studies, the positive effects of good quality direct contact and extended contact were explained by reduced intergroup anxiety: the better the quality of contact that young people have with older adults and the more intergenerational relationships they are aware of, the less anxious they are about impending intergenerational encounters, and the less ageist they are. Finding that intergroup anxiety mediates the effects of direct contact is consistent with previous intergenerational contact studies (Bousfield & Hutchison, 2010; Hutchison *et al*., 2010) and the wider contact literature (for a review, see Pettigrew & Tropp, 2008). However, the present studies are the first to demonstrate that intergroup anxiety also mediates the effects of extended intergenerational contact. These findings suggest that like other types of prejudice, ageism can be appropriately conceptualized as an intergroup process (Fox & Giles, 1993).

In Study 2, the relationships between both contact quality and extended contact and reduced ageism were also mediated by reduced ageing anxiety. This suggests that positive direct or indirect experiences with older adults have the potential to reduce the concerns that young people may have about their own ageing, and therefore to improve their attitudes towards older people as a whole. While previous research has indicated that direct intergenerational contact can reduce ageism indirectly by reducing ageing anxiety (Allan & Johnson, 2009; Allan *et al*., 2014), the present studies are the first to show that extended contact can also reduce ageing anxiety and therefore indirectly reduce ageism.

In Study 3, contact quality and extended contact were similarly associated with less ageing anxiety, and ageing anxiety was associated with less ageism, but the indirect path from contact quality and extended contact to reduced ageism via ageing anxiety was not significant. Finding that ageing anxiety mediated both direct and extended contact's relationships with attitudes in Study 2 but not in Study 3 may again be attributable to differences in the samples used. In particular, university students, who formed a higher proportion of the sample in Study 2 than Study 3, are likely to have more direct and extended contact with competent older adults (e.g., professors, mature students), which may help explain the stronger correlations between both types of contact and ageing anxiety in Study 2 than in Study 3, and hence the lack of mediation effects in Study 3. Future research should measure the competency and/or dependency levels of older adults involved in intergenerational contact and examine their relationships with ageing anxiety (see also Allan & Johnson, 2009).

At face value, finding that intergenerational contact reduces ageing anxiety and indirectly reduces ageism may appear to contradict the idea that older adults present a threat to young people by reminding them of their own ageing (Greenberg *et al*., 2002). However, rather than being contradictory, it seems reasonable to assume that positive direct or indirect experiences with older adults may go some way towards disconfirming the negative expectations young people may have about older adults and the ageing process more generally, therefore reducing their concerns about their own ageing and improving their attitudes towards older people as a whole. Along these lines, Hutchison *et al*. (2010) found that good quality contact with older adults was associated with more positive expectations among young people about the possible outcomes of intergenerational encounters, which in turn predicted less ageism (see also Plant & Devine, 2003). Future research should investigate the role of young people's expectations about the consequences of ageing in the relationship between intergenerational contact, ageing anxiety, and ageism.

In Study 2, we also examined ingroup norms and self‐disclosure as potential mediating variables, finding that ingroup norms mediated extended contact's reduction of ageism. This is consistent with the idea that experiencing indirect cross‐group friendships increases their acceptability by making them appear more widespread and familiar, thus creating an indirect pathway to more positive attitudes (Wright *et al*., 1997). Although good quality direct contact was associated with more positive ingroup norms about intergenerational relationships, and ingroup norms was associated with less ageism, there was no indirect pathway from contact quality to attitudes via ingroup norms. Finding that ingroup norms mediate the effects of extended contact but not direct contact is consistent with previous contact studies (e.g., De Tezanos‐Pinto *et al*., 2010) and supports the idea that knowing that their same‐aged peers have older friends makes intergenerational friendships seem more widespread and acceptable, thus reducing ageism. On the other hand, having direct intergenerational friendships does not provide information about the frequency of other young adults’ intergenerational friendships or the acceptability of such friendships. This may explain why ingroup norms mediated the relationship between extended contact and reduced ageism but not the relationship between contact quality and reduced ageism.

Although self‐disclosure did not emerge as a significant mediating variable in Study 3 it was positively correlated with direct and extended contact and with more positive attitudes towards older people. This suggests that, as with other types of intergroup contact (Turner *et al*., 2007), self‐disclosure has the potential to reduce prejudice but in the context of intergenerational contact the effects are not sufficiently strong to explain the relationship between contact and more positive attitudes towards older people. One reason for this may be due to problems in young people's communication with unfamiliar older adults. For example, young people are more likely to communicate with, and therefore self‐disclosure to, other young people especially via social media, which is less commonly used by older adults (Duggan & Brenner, 2013; Walther, 1996). Additionally, young adults often feel patronized (Giles & Williams, 1994) and anxious when experiencing excessive self‐disclosure by older adults (Coupland, Coupland, & Giles, 1991). Thus, it may be that intergenerational anxiety needs to be reduced before self‐disclosure can mediate between intergenerational contact and ageism. Along these lines, Pettigrew and Tropp (2008) proposed a causal sequence whereby initial anxieties must first be reduced through intergroup contact before other variables can affectively contribute to prejudice reduction. Future research should test this sequence in the context of relations between young and older adults. It may be that self‐disclosure plays a more important role in reducing ageism once intergenerational anxieties are reduced.

Taken together, the findings suggest that designs for successful ageism reduction interventions could utilize either direct or extended contact (see Cameron *et al*., 2011; Jarrott & Smith, 2011) or a combination of both. For example, Eller *et al*. (2012) showed that extended contact paved the way for future direct contact, which in turn predicted more positive outgroup attitudes. Therefore, employing an extended contact task before a direct intergenerational programme could potentially enhance the outcomes. Alternatively, direct intergenerational programmes could be followed up by young adults’ peer‐to‐peer dissemination of their positive programme experiences, thus creating an extended contact effect with other young adults not involved in the original direct programme (see also Atkinson & Bray, 2013).

Furthermore, our findings shed light on some of the psychological mechanisms through with direct and extended contact can reduce ageism. In particular, the results suggest that important variables to consider when devising strategies or interventions aimed at reducing ageism are those that assist in the reduction of young people's anxieties about intergenerational encounters and their own ageing. It is similarly important to foster the formation of positive ingroup norms about intergenerational relationships and to encourage voluntary sharing of personal information. The present research suggests that good quality direct, and extended intergenerational contact may go some way towards achieving these aims, and strategies targeted at reducing ageism should capitalize on the findings.

Although the results from all three studies are broadly in line with predictions, there are limitations with the present research. As with most contact studies, our findings are based on cross‐sectional survey data, which makes it difficult to draw firm conclusions about causal relationships. With this in mind, future research should be conducted longitudinally and experimentally to allow for stronger inferences about the relationships between the variables examined in the present studies (e.g., Eller, Abrams, & Zimmermann, 2011).

It should also be noted that negative attitudes towards older people are often internalized in older adults themselves (Levy & Banaji, 2002). This is not entirely surprising given that young people with ageist attitudes will in time become members of the older generation. Such attitudes may reinforce the marginalization and disempowerment of older adults (Bousfield & Hutchison, 2010). Thus, future research and ageism reduction interventions should seek to identify factors influencing older adults’ ageism towards their own generation as well as the ageism of young people towards older adults.

In conclusion, the present results suggest that direct contact with older adults may not be necessary to reduce ageism in young people: simply knowing that other young people have positive relationships with older individuals may be sufficient to achieve this aim. The results also shed light on the psychological mechanisms through which direct and extended contact can reduce ageism. These findings may be important when devising strategies aimed at reducing ageism, especially in the current social climate where the gap between young people and older adults is widening and opportunities for direct intergenerational contact are becoming increasingly limited.

## Footnotes

1 To obtain a clearer picture of the unique effects of extended contact across the three studies, we conducted a meta‐analysis to examine the aggregated effect of extended contact on attitudes. This confirmed that extended contact was significantly associated with attitudes even when controlling for direct contact (contact frequency and contact quality) and demographic variables: *Z* (weighted by sample size, *N *=* *273) = 5.02, mean *R* ^2^ = .31, mean Fisher's *Z *=* *.32, *p *=* *2.67 E‐7. The fail‐safe number (*p *=* *.05) was 25.2.
